# Bacteriology

**Published:** 1901-09

**Authors:** C. N. Peirce

**Affiliations:** Philadelphia


### BACTERIOLOGY.

BY C. N. PEIRCE, D.D.S., PHILADELPHIA.

OCCURRENCES which are obscure, or their cause not immediately obvious, we term phenomena. When time and experience reveal their application to the economy of life, they are termed natural law. Not infrequently the development of the individual to unravel the mystery and demonstrate its value to present and succeeding generations is tardy in materializing.

The solar system waited many centuries for Galileo, Kepler, and Newton to satisfactorily explain its structure, operation, and complex movements.

Fabricius, Harvey, and Malpighi made generation, physiology, and anatomy of the human body comprehensible by years of patient study.

Franklin, by interrogating the lightning in the clouds, made electricity the servant of man, which, by the transmission of thought, annihilates space and time.

Buckland, Lyell, and Agassiz bore eloquent testimony to the natural but sublime history of the rocks and fossils, and the age of man and animals was greatly extended.

Cuvier, Carpenter, and Spencer elaborated the principles governing society and the trend of the simple to the complex, and established the sciences of biology and sociology.

Lamarck, Darwin, Wallace, and Huxley demonstrated descent with modification, and established the origin of species and principles of evolution.

Cope, Leidy, Le Conte, and Marsh faithfully studied laws of development, vertebrate zoology, paleontology, and comparative anatomy, and established the close relationship of animals.

Faraday and Tyndall established the movements of the glaciers, studied the drops of water, the rays of light, and proved the reign of natural law.

M. Louis Pasteur, engrossed in his experiments of crystalline faucets in the tartrates, and the paratartrates, and in the molecular symmetry and dissymmetry of crystals and the range of chemical processes apart from the play of vitality, had his attention directed to ferments by a German manufacturer of chemicals, who found that mineral substances sullied with organic matter of various kinds fermented when dissolved in water and exposed to summer heat. In this solution Pasteur found the fermentation due to the multiplication of a microscopic organism, which in the liquid found its proper aliments. In this little organism he recognized a living ferment, which on examination he believed to be analogous to the yeast-plant, the alcoholic ferment to which his attention had been directed by Schwann.

Following this, Pasteur made some remarkably successful experiments with the seeds of the common mould, *Penicillium glaucum.* This brought the noted scientist unexpectedly amid the phenomena of fermentation, and that ferments in all cases are living things was his conclusion, also that the substances formerly regarded as ferments are, in reality, the food of the ferments.

He proved the ferment of lactic acid to be an organism of one kind, that of butyric acid to be an organism of a different kind. Following this he was led to the belief that the capacity of an organism to act as a ferment depended on the solution in which it was immersed, and also its power to live without air. The fermentation of beer suggested to him this idea. The yeast-plant, like many others, can live either with or without free air. In contact with the latter it is spared the labor of wrestling from the malt the oxygen required for its sustenance. He finally divided these microscopic organisms into two great classes, which he named, respectively, aërobies and anaërobies, the former requiring free oxygen to maintain life, the latter capable of living without free oxygen, but able to wrest this element from its combination with other elements.

It is to Schwann, however, that the scientific world is indebted for the first successful effort in sterilization. He placed decoctions of meat in flasks, boiled them, then supplied them with calcined air, the power of which to support life was unimpaired, yet fermentation or putrefaction in these vessels never occurred. Hence the legitimate conclusion was that putrefaction was not due to contact with air, but to something suspended in the air which heat would destroy. This something consisted of living organisms, which nourished themselves at the expense of the organic substance, and caused its putrefaction.

The acetic fermentation was next studied, which was found to be the result of a minute fungus, the *Micoderma aceti.* By this the sugar of the grape-juice is transformed into carbonic acid and alcohol, the latter remaining in the wine. The manufacture and maladies of wine occupied his serious attention. Each of the disorders of wine was traced to its specific organism, which, acting as a ferment, produced substances agreeable or otherwise to the palate. Through the influence of the illustrious Dumas, Pasteur took up the investigation of the diseases of the silk-worm at a time when this industry was in a state of ruin. Acquainted as Pasteur was with the work of living ferments, he was prepared for any emergency. Within the circulation of this insect he discovered the cause of the epidemic. The diseased corpuscles he followed through all the phases of insect life,—the eggs, the worm, the chrysalis, the moth. In the latter it reached development so distinct as to render its recognition immediate. From healthy moths healthy eggs were sure to spring; from healthy eggs healthy worms; and so on through the cycle of changes. Care in feeding and isolation of diseased from healthy worms and eggs the trouble was surmounted and the industry re-established. It was no hypothetical problem; the trouble was in a definite organism. Wherever ferments are utilized, the microscope and results must test the quality of the true torula, or yeast-plant. The germ theory of infectious diseases was the result of the researches of Pasteur and Schwann.

Pasteur was not a physician, and he did not feel himself called upon to trench upon the domain of the healing art, but by his studies and experiments he established the parasitic character of the several diseases affecting fowls, sheep, dogs, and cattle.

The work so valuable, done by Dr. Koch on splenic fever (malignant pustule), with the life history of *bacillus anthracis,* the contagion of this fever, is but a service in honor of Pasteur. The recital of the foregoing evidences of increasing knowledge and demands of the age have daily utilized all that has been evolved, until the Philosopher's stone and the transmutation of metals are relics of the past.

Alcoholic fermentation was for many years supposed to be the seat of active, spontaneous, self-regulated change; its results were known, but its origin and processes had not been revealed. The chemist assumed that the decomposition which took place was due to the aggressive action of the oxygen of the air, and the preventive process was simply its exclusion.

On the publication of Pasteur's work this spontaneous idea entirely passed out of mind, though it had been accepted as a fact even by scientific men who held that it was the simplest explanation of the origin of the swarms of microscopic organisms observed in putrefying liquids or infusions. When we recall the universal ignorance concerning the nature and complexity of various forms of life, and note the utter unreliability of any theory regarding them, it is not surprising that abiogenesis should have been held so firmly in favor and with so much confidence.

Yet Leeuwenhoeck, a microscopist of marked ability for his time (the seventeenth century), held that the entire absence of abiogenesis in the higher forms of life made it very improbable that the lower or even lowest should have such an origin; these conclusions, while not based on any recognized scientific evidence, were certainly wise, even though intuitive or instinctive.

The germ theory of disease has done much towards relieving the world of a sorry superstition. Pestilences, epidemics, plagues, are now, with scarlet fever, measles, small-pox, whooping-cough, and numerous diseases with which the human family are afflicted, looked upon as due to natural causes rather than the visitation of an angry God.

Bacteriology has been defined as a subdivision of microbiology and as the science of the culturable micro-organisms, but it must not be inferred that all such organisms are harmful to more complex or highly organized beings.

The number that are not only benign but quite essential to the life of man are greatly in excess of the harmful or malignant type. It would be difficult to conceive of the condition of our environment were we but for a limited time to be deprived of the helpful though unseen activity of the myriads of these organisms.

Professor W. S. Sedgwick brings this matter clearly before his readers when he refers to their influence in the following terms: "Without their activity the habitable world and the sea would become one vast charnel-house, because there would be no adequate agency for mineralizing dead matter. . . . We have only to think of their helpful and wholesome unseen activity in removing from our view the dead animal bodies which would otherwise cover the earth, the dead leafage of the autumn, the worn-out trunks of trees, and the waste matters of human and animal life, in order to appreciate in some measure their fundamental importance in nature.

. . . When to this we add their tendency to cause the destruction of valuable organic matters, such as food and timber; their function in producing those fermentations, putrefactions, and poisonings of the human body which we know as epidemics, plagues, pestilences, infectious diseases, suppurating wounds, gangrene, and the like; when, furthermore, we consider their causative participation in such universal, familiar, and important processes as bread-making, brewing, vinegar-making, the fermentations of milk and its products, butter-making, cheese-making, lactic acid manufacture, tanning, and nitrification, we are in a position to understand something of the scope and significance of the culturable micro-organisms, and therefore of bacteriology, from a practical point of view."

With these important facts before us, it should be borne in mind that microscopic life is, in its largest influence, beneficent to humanity; that the varieties associated with disease are comparatively few in contrast with the others. While these minute organisms have been definitely identified with special diseases, many have serious doubts as to whether it has been satisfactorily demonstrated that they are the cause and not the product of the condition.

The presence of these organisms in nearly all diseases is fully recognized, but their influence, as we see, is somewhat conjectural. The query is, Will future generations modify the modern teaching?

From published reports we learn that physicians have much difficulty in establishing positively the absence of the bacilli in certain diseases, hence the embarrassment in declaring that the disease does not exist; yet their presence would be considered positive evidence of the disease. This difficulty must be a great source of confusion to the diagnostician, and must be increased by another uncertainty mentioned in their reports,—namely, that they, the bacilli, vary greatly in number within a limited time. Another serious difficulty encountered is that bacilli have been found on nasopharyngeal surfaces and on the tonsils when there has been no systemic disturbance, and also after serious trouble has subsided their presence has been recognized. If these statements are correct, they must add to the uncertainty of their influence and make them a precarious feature of a diagnosis.

The origin of bacteria and the study of bacteriology have been extremely interesting scientific features in the last thirty years. The scope, or field, is broad and valuable, offering great compensation for laborious and painstaking cultivation, but this must be done with a full appreciation of the danger of careless and hasty conclusions.

